# Can mental health interventions change social networks? A systematic review

**DOI:** 10.1186/s12888-015-0684-6

**Published:** 2015-11-21

**Authors:** Kimberley Anderson, Neelam Laxhman, Stefan Priebe

**Affiliations:** Reinier van Arkel Groep, Bethanistraat 2, 5211 LJ, ‘s-Hertogenbosch, Netherlands; Unit for Social and Community Psychiatry (WHO Collaborating Centre for Mental Health Service Development), Queen Mary University of London, Newham Centre for Mental Health, Glen Road, E13 8SP London, UK

**Keywords:** Social networks, Psychosis, Social isolation, Psychosocial intervention, Review

## Abstract

**Background:**

Social networks of patients with psychosis can provide social support, and improve health and social outcomes, including quality of life. However, patients with psychosis often live rather isolated with very limited social networks. Evidence for interventions targeting symptoms or social skills, are largely unsuccessful at improving social networks indirectly. As an alternative, interventions may directly focus on expanding networks. In this systematic review, we assessed what interventions have previously been tested for this and to what extent they have been effective.

**Methods:**

A systematic review was conducted of randomised controlled trials, testing psychosocial interventions designed to directly increase the social networks of patients with psychosis. Searches of five online databases (PsycINFO, CINAHL, Cochrane Database, MEDLINE, Embase), hand searching of grey literature, and both forward and backward snowballing of key papers were conducted and completed on 12 December 2014. Trial reports were included if they were written in English, the social network size was the primary outcome, participants were ≥ 18 years old and diagnosed with a psychotic disorder.

**Results:**

Five studies (*n* = 631 patients) met the complete inclusion criteria. Studies were from different countries and published since 2008. Four trials had significant positive results, i.e. an observable increase in patients’ social network size at the end of the intervention. The interventions included: guided peer support, a volunteer partner scheme, supported engagement in social activity, dog-assisted integrative psychological therapy and psychosocial skills training. Other important elements featured were the presence of a professional, and a focus on friendships and peers outside of services and the immediate family.

**Conclusions:**

Despite the small number and heterogeneity of included studies, the results suggest that interventions directly targeting social isolation can be effective and achieve a meaningful increase in patients’ networks. Thus, although limited, the existing evidence is encouraging, and the range of interventions used in the reported trials leave various options for future research and further improvements. Future research is needed to test the findings in different settings, identify which components are particularly effective, and determine to what extent the increased networks, over time, impact on patients’ symptoms and quality of life.

**Electronic supplementary material:**

The online version of this article (doi:10.1186/s12888-015-0684-6) contains supplementary material, which is available to authorized users.

## Background

People with psychosis frequently experience difficulties in developing and maintaining social relationships; their networks tend to be smaller than those of people without mental illness, and are largely composed of family members [1]. Over longer periods of time, people with enduring psychosis often rely on healthcare services to maintain social bonds [2].

Living alone, having few social ties and infrequent contact are all indicators of social isolation [3], and the size of social networks can be used as a measure of isolation or connectedness. Social networks include both close, supportive relationships with family and friends, as well as more spontaneous, casual interactions with wider contacts in the community. Pattinson & Pattinson [4] define social networks based on the closeness and frequency of the individual social interaction, including; personal, intimate, effective, nominal and extended zones of networks. A greater sense of independence, better overall health and fewer social stressors are all mediators of social networks, and have positively been shown to have various effects on wellbeing, including: feelings of belonging, reducing stress, restoring hope, and increasing ability to adapt to new situations [5]; instilling feelings of trust and reciprocity [6]; providing higher community functioning [7]; increasing engagement with mental health services [8], and improving quality of life [9].

Equally however, several factors play a role in limiting the networks of people with psychosis. Symptoms of the disorder, in particular negative symptoms: anhedonia, emotional dullness and low energy, are known to impair the motivation and ability to establish and maintain social relationships. Disadvantages such as unemployment and poor socio-economic status may also reduce the opportunities to utilise social skills [10].

The challenge for mental health services is to overcome these problems in order to help people with psychosis build and sustain a sufficiently large social network. The existing evidence suggests that established treatments targeting negative symptoms largely fail (see Carpenter and colleagues [11] for more detail on this), although such symptoms may change over time [12]. Thus, the evidence supporting treatments that aim to enlarge networks indirectly–by primarily targeting underlying symptomatology–is limited. We therefore conducted a systematic review of randomised controlled trials (RCTs) whose primary aim is to improve the social networks of patients with psychosis, to determine the effectiveness of existing psychosocial interventions. (Prospero ID: CRD42015020540).

## Methods

We intended to be inclusive with respect to the characteristics of patients, the type of interventions and the exact measure of social networks. Yet, the review was restricted to studies that assessed some behavioural indicator of social networks as primary outcome. We did not consider patients’ satisfaction or other forms of subjective appraisal as an outcome, since such appraisals are only moderately linked with objective measures of social networks. The restriction to studies with social networks as primary outcomes enabled us to avoid bias through including studies that would selectively report secondary outcomes, and possibly over-report outcomes showing a positive effect of the given intervention.

### Eligibility criteria

Included studies were required to have a primary outcome measure of social network size, and were excluded if only broader reference to social interactions was made, or social network size was a secondary outcome measure. Studies were included if participants were recruited from inpatient, outpatient or community mental health care services; ≥ 50 % participants with psychosis, and at least 18 years of age. Treatment as usual, active and wait-list control conditions were all accepted as comparators. There was no restriction on publication date, but due to resource constraints we excluded those not written in English. Reviews were excluded but their reference lists were screened.

### Search strategy

A systematic, electronic search was conducted on the databases PsycINFO, MEDLINE, CINAHL, Embase and the Cochrane Library, and publication bias was minimised by including conference papers and book chapters, searching grey literature, and corresponding with authors to identify additional work where necessary. Reference lists of key studies and identified reviews were searched and relevant papers obtained (backward snowballing), as well as finding citations to studies documented (forward snowballing). The search was completed on 12 December 2014, and was conducted by researcher KA. A hand search for relevant articles in key journals, such as *Schizophrenia Bulletin*, *British Journal of Psychiatry*, *Psychiatric Bulletin* and *Archives of General Psychiatry*, was carried out by NL. Using a random-number generator, a 20 % check verified eligibility of included and excluded abstracts.

The combination of terms was deliberately broad to increase sensitivity of the search and identify all eligible RCTs. The search strategy was defined as terms containing adjectives or derivatives of ‘psychosis,’ ‘social networks’ and ‘intervention’ that were combined using a series of boolean ‘AND/OR’ operators (see Additional file 1). Specific named interventions were included following their identification in key texts. Data extraction and quality assessment was guided by The Cochrane Collaboration’s Intervention Review for RCTs, and each eligible full-text study was double rated by researchers KA and NL. Disagreements were discussed with SP and consensus was reached on the final inclusion of studies.

### Data analyses

General characteristics of the studies and participants were extracted for each trial, as well as details on the intervention groups, outcomes, risk of bias assessments and data analysis. Trends in social network size before and after the interventions were planned to be analysed by pooling the data, and comparing standard mean differences. However, because of the small number and high heterogeneity of studies, a meta-analysis was prevented. Instead, findings were reported narratively.

## Results

### Results of the search

**Fig. 1:**
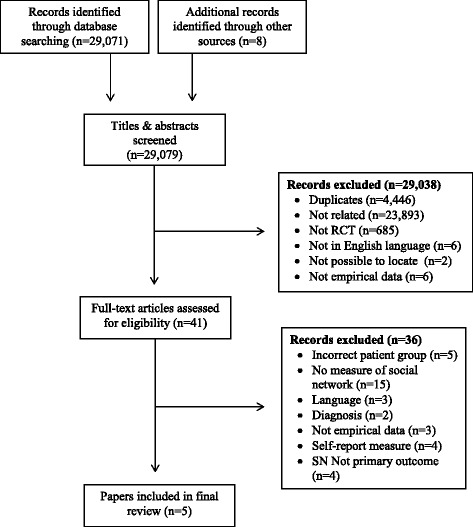
PRISMA Flow diagram for paper selection

The search produced a total of 29,079 titles to screen, and after removing duplicates and irrelevant papers; a full text assessment of 41 documents was conducted. Five trials with a total of 631 patients met the exact inclusion criteria, and the PRISMA [13] flowchart in Fig. 1 depicts the screening process and exclusion of papers.

The five included studies were conducted in: Italy (*n* = 347 participants), Ireland (*n* = 102), Netherlands (*n* = 106), Spain (*n* = 21) and Israel (*n* = 55), and they span a publication period from 2008 to 2014. There was a total of 436 males (69 %) and a mean age of 44.2 (SD 6.0) years. Three studies took place in community mental health settings [14–16], one in a mental health centre (which was neither identified as inpatient nor outpatient services) [17], and one in an inpatient facility [18]. All studies sought and obtained ethical approval and patient consent to participate.

Two studies [15, 16] assessed the diagnosis of participants using ICD-10 criteria [19], two [18, 19] used DSM-IV [20], and one study [16] did not provide any information on the diagnostic system. Two studies [15, 19] featured diagnoses of ‘schizophrenia,’ one [17] ‘psychosis, one [15] featured ‘severe mental illness’ accepting patients with F20-F49 ICD-10 diagnoses, and one [16] specified ‘serious mental illness’ accepting diagnoses of schizophrenia, schizoaffective disorder, depression and bipolar disorder. The specific duration of illness for participants in these studies was unable to be ascertained, since reporting on this detail was insufficient, but most report chronic patients. All interventions took place on a weekly basis, with an average number of 1.8 (SD .75) hours per week, and with an average study follow-up period of 11 months (SD 8.7). All studies intended to improve contacts outside of the family. Table 1 gives an overview of included studies.

**Table 1:** Summary of study characteristics

Study	Country	No. participants	Setting	Diagnostic criteria	Disorders featured	Intervention outline	Control	Duration of intervention	Follow-up period
Terzian et al. [14]	Italy	172: Intervention, 173: Control	Community	ICD-10	Schizophrenia	Staff identified possible areas of interest for patients to take place outside the services’ resources and with members of the community at large.	Routine care	6 months	2 years
Sheridan et al. [15]	Ireland	52: Intervention, 55: Control	Community	ICD-10	Schizophrenia	Patients matched with volunteer + €20 monthly stipend for activities.	Stipend only	2 h a week for 9 months	9 months
Castelein et al. [17]	Netherlands	56: Intervention 50: Control	Mental Health Centres	DSM-IV	Psychosis	Guided peer support group with focus on social network, social support, self-efficacy and quality of life.	Wait list	16 sessions for 90mins each over 8 months	8 months
Hasson-Ohayon et al. [16]	Israel	33: Intervention, 21: Control	Community	Insufficient information	Schizophrenia, schizoaffective disorder, depression, bipolar disorder	Social Cognition Interaction Training (SCIT): Social, leisure, support, employment services + social mentoring	Social mentoring	1 h sessions 3 × per week	Insufficient information
Villalta-Gil et al. [18]	Spain	12: Intervention, 9: Control	Inpatients	DSM-IV	Schizophrenia	Integrated Psychological Therapy (IPT) improving social and cognitive functioning, social perception, problem solving, verbal communication + ‘therapy dog’ present in sessions	IPT	45 minute sessions 2 × per week	12.5 weeks

### Characteristics of interventions

Overall, the structures of the interventions tested in these studies are highly diverse. In one trial conducted in Italy [14], participants worked closely with clinical staff to identify possible areas of interest and activities they might like to take part in. This involved forming social contacts in the community; outside of their family, mental health services and other patient groups. Participants were encouraged and supported by staff to engage in these activities. Meetings were once a week for an hour over a period of 3–6 months.

Another study, conducted in Ireland [15], matched participants with volunteer partners to facilitate social connectedness. The authors attempted a replication of a trial previously conducted in the USA [21] (which was excluded from this review since their primary outcome was not social network size) and adapted their study to include a stipend for one arm and an explicit measure of social network size. However, due to recruitment issues, they were only able to randomise to two arms instead of three (volunteer + stipend, and stipend only). The volunteer-participant pairs met for 2 h per week, and spent time in the community together doing activities of interest to the patient.

The Dutch trial in this review [17] delivered guided peer support, which involved a nurse facilitating a talking group for participants with psychosis, but which was primarily directed by the members. The group offered a structured and continuous discussion of issues related to mental illness. The presence of a nurse has previously been noted as helpful for patients to develop a sense of security and meaningfulness, while promoting group interactions without actively affecting the non-specific group processes.

The study conducted in Israel [16] tested social cognition and interaction training (SCIT), whereby participants worked in groups to explore impairments in their social cognitions. In addition, both the experimental and control groups received ‘social mentoring’ which involved weekly meetings with an individual mentor to set realistic goals related to patients’ social life, such as organising their finances, enrolling on a course or engaging with the community in some way.

The final study in this review, conducted in Spain [18], used integrative psychological therapy (IPT) as their main care approach, which is a structured group intervention with five distinct elements: cognitive differentiation, social perception, verbal communication, social skills training, and interpersonal problem solving. They additionally combined this with the presence of a ‘therapy dog’ to the experimental arm, as they reported that animals can have positive effects on social functioning, and participants were encouraged to engage and interact with the dog during sessions.

Control groups were variants of routine care as the main comparator. This typically involved usual contact with a person’s regular care team, maintaining scheduled appointments, adhering to medication as required or attending groups as per their normal programme.

### Effects of interventions

Four out of five studies [14, 16–18] in this review successfully report significant increases in social network size. This equates to a 45.5 % increase in social network size for the Italian study [14], who found no further statistically significant improvements in their secondary outcomes: clinical information, activities of daily living and work; and a 56 % increase for the Dutch study [17], who also indicate significant improvements in self-esteem and quality of life for those who regularly attended their peer support groups. For the Israeli study on SCIT, a significant, albeit small, increase in interpersonal communication scores at the end of the intervention was found (2.9 % increase), and Vilalta-Gil and colleagues demonstrated a significant positive increase in the social contact score of the Living Skills Profile (*p* = .041). Lastly, Sheridan and colleagues in Ireland [15] indicated a trend towards increased social network size at the end of the intervention period (2 years)–a 7 % improvement–but these results were not found to be statistically significant. Although overall most outcomes indicated trends towards symptom reduction and increases in personal strengths, only those cited were statistically significant. It was not possible to ascertain what these figures represent in terms of size of social network (see Additional file 2 for a summary).

### Social network assessment tools

A wide variety of assessments were used to measure social networks across the five trials; in fact no two trials used the same tool. Three trials used explicit counting methods to measure number of contacts within participants’ social network, and of these only the volunteer partnership study [15] used a validated tool–the Practitioner Assessment of Network Type [22]. Although the other two did not use established measures to assess the network size, they were similar in their counting methods. The guided peer support study [17] used the Personal Network Questionnaire (PNQ), a self-developed measure to record frequency and importance of relationships; and the social activities study [14] had a basic scoring system for the type of relationships and the frequency of encounters. For these studies, higher scores were interpreted as more contacts/greater social network.

The further two trials [16, 18] used relevant subscales within validated measures. The Social Functioning Scale (SFS) [23] and Living Skills Profile (LSP) [24] respectively, are both tools designed specifically for patients with schizophrenia. For the SFS, ‘interpersonal communication’ was used in this instance to measure the size of the participants’ social network and the ability to effectively interact with others. For the LSP, higher scores within the ‘social contact’ item also indicate better social engagement. Although these do not ‘count’ the number of contacts in the same way the previous measures do, they are be considered to capture the social network size.

### Quality of included studies

Overall, the studies included in this review did not indicate high risk of bias, although there appear to be some problems with the quality of reporting, and with rigorous blinding.

All except the Israeli SCIT study [14, 15, 17, 18] were allocated to treatment arm by automated computer or telephone service, and were rated as having a low risk of randomisation bias. All studies took precautionary measures to ensure the assignment of participants to treatment arms was not affected by selection bias: including balancing allocation by telephone according to site, using independent personnel to carry out randomisation, using sealed envelopes and weighted recruitment sites so each participant had equal chance of being randomised to experimental group. Blinding of participants across trials was generally not employed, likely due to the nature of psychosocial interventions whereby participants are aware of new or additional care approaches they are part of.

The Italian social activities and Irish volunteer partnership studies [15, 16] were rated as high risk of bias for blinding of assessors. Reasons for this include the absence of blinding procedures overall, revelation of group allocation by participants during data collection, or failing to blind independent personnel. For missing outcome data, only the Italian social activities and Dutch guided peer support studies [15, 16] reported how missing data was dealt with. The remaining studies failed to convey why missing data occurred or how this was managed in their analyses, and therefore no judgement could be made. Table 2 provides an overview of this.

**Table 2:** Summary of the risk of bias using the Cochrane Collaboration’s Intervention Review for RCTs

Study	Random sequence generation	Allocation concealment	Blinding of participants	Blinding of assessor	Incomplete data outcome	Selective reporting
Terzian et al. [14]	Low risk	Low risk	High risk	High risk	Low risk	Low risk
Sheridan et al. [15]	Low risk	Low risk	High risk	High risk	High risk	Low risk
Castelein et al. [17]	Low risk	Low risk	Low risk	Low risk	Low risk	Low risk
Hasson-Ohayon et al. [16]	Unclear risk	Low risk	Unclear risk	Unclear risk	High risk	High risk
Villalta-Gil et al. [18]	Low risk	Low risk	Low risk	Low risk	Unclear risk	Low risk

## Discussion

This review examined interventions to improve social networks for patients with psychosis, and assessed the effectiveness of these. To our knowledge, no review has previously explored this. It was achieved through a detailed systematic search of existing literature, and all available randomised controlled trials with an objective primary outcome measure of social network size were included. In total, only five studies met the specific inclusion criteria–albeit with a substantial total participant sample size of 631. Four out of the five studies reported statistically significant improvements in the social networks of patients following their interventions. It may be concluded that despite some recent therapeutic pessimism with regard to improving social outcomes for this patient group [25], interventions directly targeting social connectedness appear promising.

Nevertheless, the small number (more than half of the included patients were from one multi-centre trial in Italy), and heterogeneity of studies, with no two studies testing similar interventions, limits the robustness of the conclusions. Equally, the heterogeneity of assessment tools makes comparison difficult, a concern of social network measures captured previously by Gayer-Anderson & Morgan [26].

### Interpretation

Aspects of guided peer support, community participation and engagement, skills training and animal-assisted IPT feature across the interventions in the positive trials, whereas a volunteer partnership scheme had less favourable results. This suggests that different interventions, including some that are potentially not very intensive or costly, might have a similar effect and help to promote and foster social interactions that constitute a social network. The presence of a professional within the interventions, regardless of their specific defined role, appears to be an important factor for increasing social networks. These professionals may act as potential mediators between formal interactions with healthcare services and more casual links in the community.

The included studies featured mostly patients with a duration of illness of several years, and the target groups were those who had already lost relationships and often lived rather isolated. The findings of the review suggest that interventions may still be helpful with such patients, and that long periods of isolation are not necessarily a reason for pessimism as to whether patients’ networks can be increased. However, one may also speculate as to whether supporting patients’ ability to maintain social networks at an earlier stage of their illness–e.g. though interventions as included in this review-might be effective in avoiding social isolation later on.

Whilst the consideration of families varied across the interventions, all of them aimed to improve contacts outside of the family. Treatments of patients with psychosis traditionally emphasise family links, e.g. though psycho-education programmes with key relatives and family therapy. Yet, patients might benefit from interventions with a broader remit that help to establish and maintain wider contacts in the community. It may be more feasible to work on new contacts rather than changing family relationships and improving already existing friendships after many years of illness.

All studies were published since 2008, suggesting a recent and possibly increasing interest in such social interventions in the scientific community. The interest might reflect a disappointment about the effectiveness of more conventional pharmacological and psychological interventions in the treatment of patients with psychosis and a search for new holistic approaches. The recent publication of these trials might also indicate that social networks have become more interesting as a target for interventions and as treatment outcomes. Future research can add to the findings reported in this review if further trials are conducted with the social network size as the primary outcome.

The findings are encouraging and point towards avenues for future research. Replication is needed through methodologically rigorous trials. Process evaluations, explanatory trials and cost-effectiveness analyses should help to identify which components are effective, efficient and particularly acceptable to patients, and to what extent the relevant components are consistent or different across interventions. All this can inform the development of intervention models for the future, which may be advancements of the interventions considered in this review or select and combine components of different interventions to design new and even more beneficial methods for increasing networks. Longer term follow-up studies should explore whether the gains in social networks are sustained over time, what support, if any, is required from health care services for this, and whether the improved networks indeed impact on patients’ symptoms and quality of life.

### Strengths and limitations

Although the included studies were heterogeneous in various respects, they all met the inclusion criteria; particularly using a behavioural measure of the social network size as the primary outcome. The number of trials is small, despite the substantial total number of patients of more than 600. The fact that the trials are from different countries, settings and tested very different interventions may be seen as a limitation. Yet, four out of these five studies yielded a significant positive finding, which may indicate that improvements of social networks can indeed be achieved with a range of interventions and in different contexts.

However, limitations of note are that only papers written in English were considered; that no meta-analysis could be performed; that we included studies with insufficient reports of study details; and that none of the studies reported an economic analysis of the costs and benefits of the intervention. Nevertheless, the limitation to studies using social network size as the primary outcome avoids reporting bias, which would have been inevitable with the inclusion of studies using social networks as one out of many secondary outcomes.

## Conclusions

Large numbers of people with psychosis remain isolated, disengaged from local activities, and socially withdrawn for long periods. The extent to which mental health services in different countries regard improving the social network of patients as a core task is likely to vary. Nevertheless, this review suggests that such improvements are possible when social activities are targeted directly rather than indirectly through symptom control.

## Additional files

Additional file 1:
**Complete list of search terms.** (DOCX 64 kb) Additional file 2:
**Summary of reported assessment & outcome data.** (DOCX 83 kb)

## Abbreviations

DSM: diagnostic & statistical manual of mental disorders
ICD: international classification of disease
IPT: integrative psychological therapy
LSP: living skills profile
PNQ: personal network questionnaire
PRISMA: preferred reporting items for systematic reviews and meta-analyses
RCT: randomised controlled trial
SCIT: social cognition and interaction training
SFS: social functioning scale
