# Supplementary material for: Can mental health interventions change social networks? A systematic review
Source: BMC Psychiatry. 2015 Nov 21;15:297. doi: 10.1186/s12888-015-0684-6 (PMC4654911; doi:10.1186/s12888-015-0684-6)
Supplement: Additional file 1: — Complete list of search terms. (DOCX 64 kb) [file 12888_2015_684_MOESM1_ESM.docx]

| **Group 1** | **AND Group 2** | **AND Group 3** |
| --- | --- | --- |
| psychosis OR | social network* OR | intervention* OR |
| psychotic disorder OR | social contact* OR | intervention study OR |
| mental dis* OR | social activit* OR | psychosocial interv* OR |
| schizophrenia OR | social isolation OR | social outcome OR |
| psychiatric dis* OR | social inclusion OR | social intervention OR |
| psychiatr* OR | social support OR | peer support programme OR |
| schiz* OR | interpersonal support OR | peer support OR |
| severe mental illness | social relationship* OR | motivational interviewing OR |
|  | close person OR | social skill* OR |
|  | social network analysis OR | social skills training OR |
|  | weak ties OR | befriend* OR |
|  | strong ties OR | vocational training OR |
|  | social ties OR | goal setting OR |
|  | social functioning | personal skill* OR |
|  | social interaction OR | life skill* OR |
|  | global network | relationship building OR |
|  |  | interpersonal skill* OR |
|  |  | problem solving skills OR |
|  |  | resource finding OR |
|  |  | supported sociali?ation OR |
|  |  | communication skill* OR |
|  |  | interpersonal communication |
