# Supplementary material for: Can mental health interventions change social networks? A systematic review
Source: BMC Psychiatry. 2015 Nov 21;15:297. doi: 10.1186/s12888-015-0684-6 (PMC4654911; doi:10.1186/s12888-015-0684-6)
Supplement: Additional file 2: — Summary of reported assessment & outcome data. (DOCX 83 kb) [file 12888_2015_684_MOESM2_ESM.docx]

| **Study** | **Time points measured** | **Secondary outcome(s)** | **Social network measure** | **Reported outcome** | **Reported data** |
| --- | --- | --- | --- | --- | --- |
| **Terzian et al. (2013)** | Baseline & 6 months | BPRS/GAF, ADL, work | Each relationship described by the patient was summarized by a score; all social contacts were quantified as the sum of all individuals | *Intervention* Year 1: 39.9% improvement Year 2: 45.5% improvement *Control* Year 1: 25% improvement year 2: 31.5% improvement | Year 1:  OR 2.0, 95% CI 1.3 to 3.1; AOR 2.4, 95% CI 1.4 to 3.9 Year 2: OR 1.8, 95% CI 1.1 to 2.8; AOR 2.1, 95% CI 1.3 to 3.5 |
| **Sheridan et al. (2014)** | Baseline & 2 years | Loneliness, self-esteem, depression | Practitioner Assessment of Network Type (PANT: Wenger & Tucker, 2002) This 8-item instrument identified the core group of people that participants in the study relied on for advice, help and support. The instrument identifies social networks as individuals, family and communities who are involved with the person in a significant way. These networks include members of the person’s household, the provision of companionship and friendship, emotional support and help from individuals and the community. | *Intervention*  Baseline contact with friends per week: 15.4%  Endpoint: 21.9%  *Control*  Baseline contact with friends per week: 18.2%  Endpoint: 25.6% | From the author *“mean/SD not calculated for PANT scale”* |
| **Castelein et al. (2008)** | Baseline, 8 months | Social support, self-esteem, confidence, QoL | Personal network questionnaire (PNQ: self-developed)  Frequency of contacts with named family, friends, and members of the peer support group. Higher # = more contacts | *Intervention* 56% increase in peer contact *Control* 31% increase in peer contact | P<.03 @ 8 months |
| **Hasson-Ohayon et al. (2014)** | *Insufficient information* | Emotion recognition, theory of mind, attributional style, | Social Functioning Scale (SFS: Birchwood et al, 1990) The social-engagement subscale measures how much time each day a person spends in the presence of others and the tendency to engage others, including strangers, in conversation. Possible scores range from 0 to 15. The interpersonal-communication subscale measures the size of each participant’s social network and the ability to effectively interact with others. Possible scores range from 0 to 9. Higher scores indicate better engagement and interpersonal communication. | Interpersonal communication scores *Intervention* Baseline mean 6.8 (SD1.8)  Follow-up mean 7.0 (SD 1.8) *Control* Baseline mean 7.1 (SD 1.4) Follow-up mean 7.1 (SD 1.3) | Intervention= 2.9% Control = No change |
| **Vilalta et al. (2009)** | Baseline & 6 months | Symptomatology, QoL | Living skills profile (LSP: Rosen et al, 1989) Higher scores relate to better functioning/more contacts | Patients in the IG+D group showed significant improvements in the LSP (social contact score) | P = 0.041 |
